# An efficient transient expression system for gene function analysis in rose

**DOI:** 10.1186/s13007-017-0268-1

**Published:** 2017-12-22

**Authors:** Jun Lu, Mengjuan Bai, Haoran Ren, Jinyi Liu, Changquan Wang

**Affiliations:** 1 College of Horticulture, Nanjing Agricultural University, No. 1 of Weigang, Nanjing City, 210095 Jiangsu Province People’s Republic of China; 2 Key Laboratory of Landscaping of Ministry of Agriculture, Nanjing Agricultural University, Nanjing city, 210095 People’s Republic of China

**Keywords:** Roses, *Agrobacterium*, Transient transformation, Shoots in vitro

## Abstract

**Background:**

Roses are widely used as garden ornamental plants and cut flowers. *Rosa chinensis* cv ‘Old Blush’ has been used as a model genotype in rose studies due to its contribution to recurrent flowering and tea scent traits of modern roses. The deficiency of efficient genetic transformation systems is a handicap limiting functional genetics studies of roses. *Agrobacterium*-mediated transient transformation offers a powerful tool for the characterization of gene function in plants.

**Results:**

A convenient and highly efficient *Agrobacterium* mediated genetic transformation protocol using *R. chinensis* cv ‘Old Blush’ seedlings in vitro as an expression system is described in this paper. The most important factor affecting transformation efficiency in this system is seedling age; 3/4-week-old rose shoots with or without roots from sub-culturing are optimal for transformation, requiring no complicated inoculation media, supplements, or carefully tuned plant growth conditions. This transient expression system was successfully applied to analysis of the gene promoter activities, DNA binding capacity of transcription factors, protein–protein interaction in physiological contexts using luciferase as a reporter gene.

**Conclusions:**

This transient transformation system was validated as a robust and efficient platform, thus providing a new option for gene function and signaling pathway investigation in roses and further extending the utility of *R. chinensis* cv ‘Old Blush’ as a model plant to study diverse gene function and signaling pathways in Rosaceae.

**Electronic supplementary material:**

The online version of this article (10.1186/s13007-017-0268-1) contains supplementary material, which is available to authorized users.

## Background

The genus Rosa comprises about 200 species, among which only 8–20 species have contributed to the genetic make-up of our present cultivars [1]. *Rosa chinensis* cv ‘Old Blush’ is one of the important progenitors of modern rose cultivars and regarded as the main contributor of some major traits, like recurrent flowering and components of the characteristic ‘tea scent’ of modern roses. *Rosa chinensis* cv ‘Old Blush’ has therefore has been proposed as a model genotype to represent the group of Chinese roses in phylogenetic studies, petal genomics and scent production analysis [1–3]. In recent years, molecular and genomic approaches have been used in roses to study the mechanisms regulating several ornamental traits such as pathogen resistance, continuous flowering, scent biosynthesis, flower color, and architecture [4]. In these studies, genetic transformation is an important tool for analysis of gene function. However, many rose cultivars are known to be recalcitrant to regeneration and hence to genetic transformation. Only a limited number of successful rose transformation protocols using somatic embryos have been reported [5, 6]. Thus, the deficiency of efficient genetic transformation systems is a handicap limiting functional genetics studies in roses.

Although stable integration of physiologically active and regulated transgenes is the ultimate goal, production and screening of stably transformed transgenic plants is time consuming even in the model plant *Arabidopsis*, where transformation techniques are well-established. *Agrobacterium*-mediated transient transformation offers a powerful alternative procedure for the characterization of gene function in plants, including analysis of gene promoter properties, transcription factor (TF) activity, and protein–protein interactions [7–11]. This method has been developed for a wide range of plants including Nicotiana, lettuce, tomato, and Arabidopsis [8–11]. The leaf infiltration assay in *Nicotiana benthamiana* is a well-established and commonly used platform for transient gene expression due to its simplicity and consistency [8]. However, in studies of diverse plant species including roses, use of *N. benthamiana* as a heterologous expression system might lead to misleading results.

The VIGS (virus-induced gene silencing) system has been exploited with vacuum infiltration of *Agrobacterium* into rose petal for silencing specific genes [12, 13]. However, this approach is limited to use in petals and thus to study flower-related physiological process and biological responses. In mature rose leaves, *Agrobacterium* infiltration is extremely difficult because of the stratum corneum and wax coat present on the outermost layer of leaves. Thus, achieving highly efficient and consistent transient expression in rose by mature leaf infiltration is challenging. To overcome this technical barrier, we developed a highly efficient and robust *Agrobacterium*-mediated transient expression system using young shoots in vitro as the infiltration target, which enabled functional analysis of diverse genes in rose. Roses have both seasonal and perpetual flowering cultivars. Recent studies have shown that the *Arabidopsis TFL1* homolog, *RoKSN*, is a major floral repressor which causes seasonal flowering and that mutation of this gene leads to perpetual flowering [14]. To confirm the biological function of *RoKSN*, we utilized our newly established transient transformation system to analyze *RoKSN* promoter and protein LUC fusions, thus demonstrating the versatile applicability of this method for examining promoter activities, transcription factor (TF) activity, and protein–protein interactions in physiological contexts.

## Results

### Impact of seedling age on transient expression efficiency

To start with the LUC activity analysis in *Agrobacterium*-infiltrated rose seedlings, we chose 20-d old seedlings and continuously took picture every 10 min for 1 h by using the CCD camera, as shown in Additional file 1: Fig. S1 the LUC activity kept stable after the second picture, so we selected the third picture for LUC activity measurement in all the followed experiments.

**Fig. 1:**
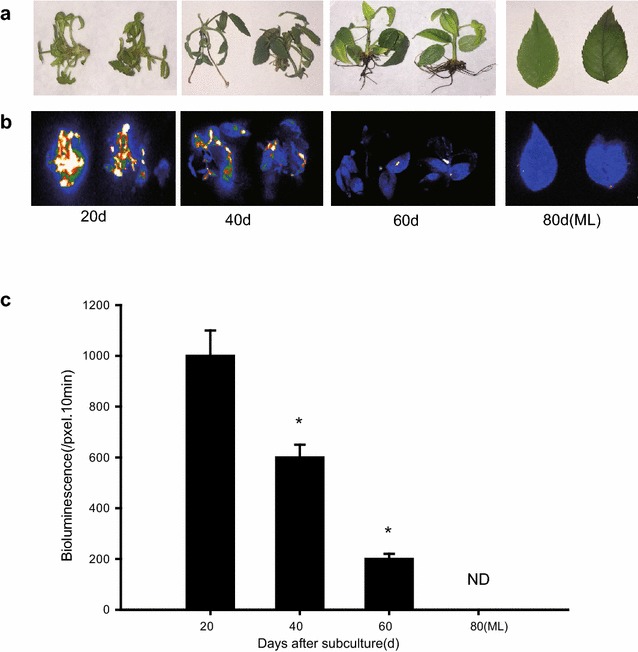
The effect of shoot age on the transient expression efficiency of roses. The fresh cut adventitious buds were transferred on proliferation medium and cultured for 3 weeks, and then transferred to rooting medium and grown for another 3 weeks to develop roots, the seedlings with roots were then planted in soil. Sub-culture times indicated in the figure refer to the days from the first transfer until LUC analysis. **a** Bright-field, **b** dark-field, **c** intensity of LUC bioluminescence quantified using Andor Solis image analysis software. Data are mean ± SEM of five biological replicates each with three technical repeats, 20 shoots were used in each technical repeat. Asterisks denote a significant *P* < 0.05

Establishing the range of shoot ages amenable for efficient transient expression is crucial for this system. We therefore tested rose shoots of different sub-culture time infected by *Agrobacterium tumefaciens*. We observed an inverse relationship between sub-culture time and LUC signal—the highest level of LUC expression was obtained with the shortest sub-culture time (20 d) and decreasing expression was observed as culture times increased, until becoming undetectable in 80 d seedlings, in which the leaf is almost mature (Fig. 1). Interestingly, the LUC signals also appeared in roots of young shoots (Additional file 2: Fig. S2).

Based on visual inspection, the lack of LUC expression in mature leaves may be due to unsuccessful infiltration, potentially caused by the stratum corneum and wax coat present on the outermost layer of mature leaves. Thus, the use of younger shoots for vacuum infiltration may be more desirable for maintaining higher expression efficiency, with a sub-culture time of 3–4 weeks being optimal for infiltration.

### Effect of infiltration buffer on transient expression efficiency

**Fig. 2:**
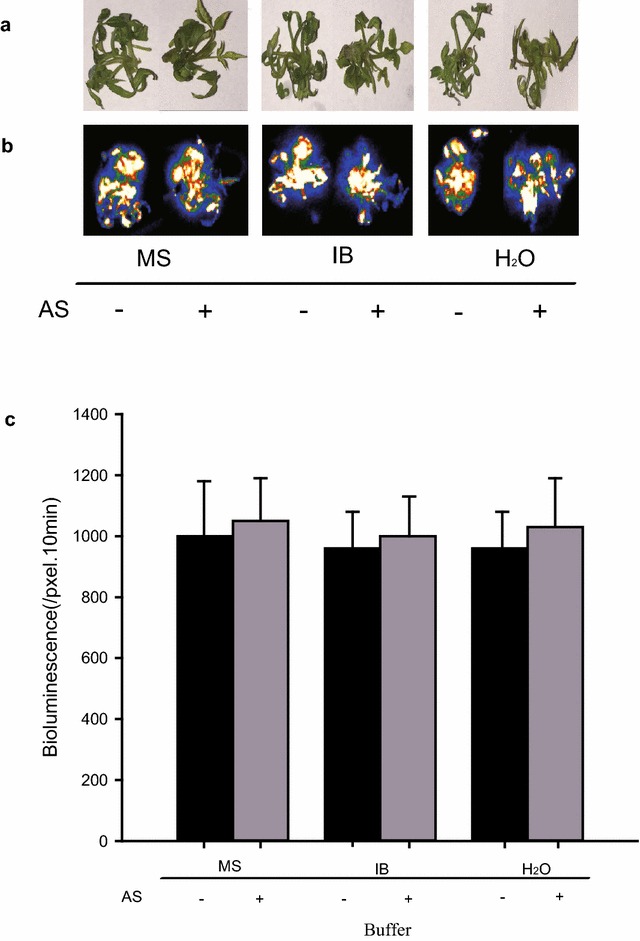
The effect of different infiltration buffers on transient expression efficiency in roses. 20-d old shoots were infiltrated with Agrobacterium carrying *pKSN:LUC* suspended with MS medium, infiltration buffer (IB) (2 mM Na_3_PO_4_, 50 mM MES, 0.5% glucose), and sterilized water with/without 100 μM acetosyringone. **a** Bright-field, **b** dark-field, **c** intensity of LUC bioluminescence quantified using Andor Solis image analysis software. Data are mean ± SEM of five biological replicates each with three technical repeats, 20 shoots were used in each technical repeat

To optimize this transient expression system for higher efficiency, we tested several factors including infiltration buffer, acetosyringone (AS) and other supplements. The commonly used infiltration medium (2 mM Na_3_PO_4_, 50 mM MES, 0.5% glucose, and 100 μM acetosyringone) for *Nicotiana benthamiana* proved to be compatible with the rose system. Interestingly, both simplified MS medium and sterilized water were found to have almost the same expression activity of LUC compared with the standard buffer (Fig. 2).

Based on previous reports that addition of AS during the infection process is required to stimulate vir genes (encoded by Ti plasmid) expression [15, 16], we tested whether this component is essential for transient expression efficiency. As shown in Fig. 2, addition of AS had no benefit on the expression efficiency of LUC gene in the present system.

### The role of P19 in transient expression

**Fig. 3:**
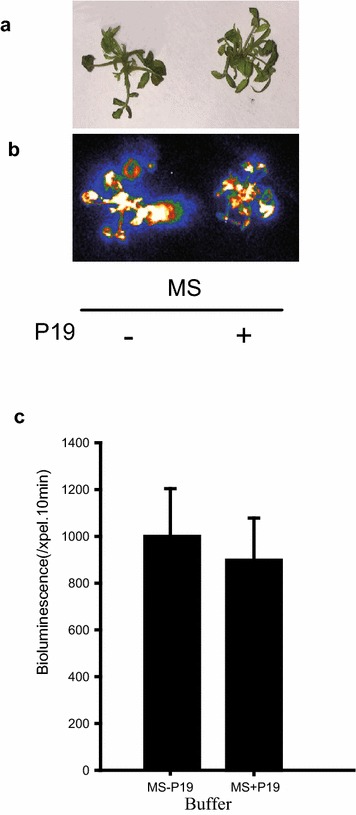
The effect of P19 on transient expression efficiency in roses. 20-d old shoots were infiltrated with Agrobacterium carrying *pKSN:LUC* suspended with MS medium with/without P19. **a** Bright-field, **b** dark-field, **c** intensity of LUC bioluminescence quantified using Andor Solis image analysis software. Data are mean ± SEM of five biological replicates each with three technical repeats, 20 shoots were used in each technical repeat

Upon transient transformation, ectopic expression of the gene of interest in plant cells usually ceases after several days due to post-transcriptional gene silencing (PTGS). It is reported that co-expression of a viral-encoded suppressor of gene silencing, the p19 protein encoded by tomato bushy stunt virus (TBSV), may prevent the onset of PTGS in infiltrated tissues, thus allowing higher levels of transient expression [17]. To test for an effect of p19 in our system, we co-infiltrated *pRoKSN:LUC* with a vector encoding p19 driven by the constitutive Cauliflower Mosaic Virus 35S promoter. Compared to a control infiltration with *pRoKSN:LUC* alone, the combination of two vectors didn’t show any improvement of LUC activity (Fig. 3).

### Promoter expression pattern and transcription factor action analysis

**Fig. 4:**
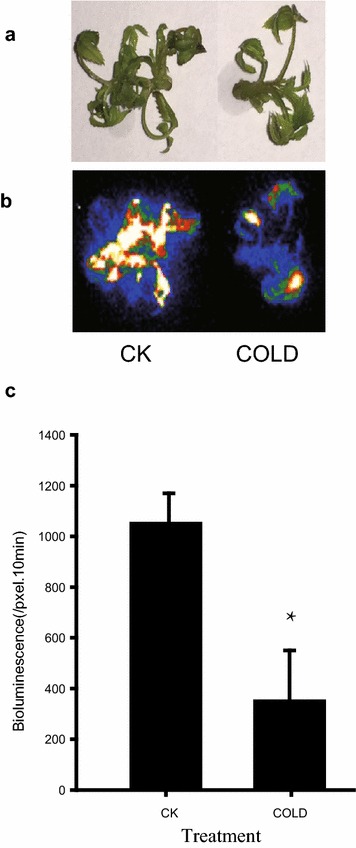
The effect of cold treatment on transient expression efficiency in roses. 20-d old shoots were infiltrated with Agrobacterium carrying *pKSN:LUC* suspended with MS medium, and kept in 4 °C for 3d before LUC analysis. **a** Bright-field, **b** dark-field, **c** intensity of LUC bioluminescence quantified using Andor Solis image analysis software. Data are mean ± SEM of five biological replicates each with three technical repeats, 20 shoots were used in each technical repeat. Asterisks denote a significant *P* < 0.05

**Fig. 5:**
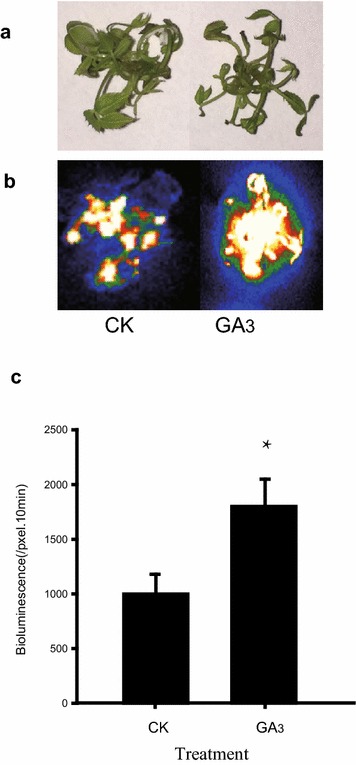
The effect of GA_3_ treatment on transient expression efficiency in roses. 20-d old shoots were sprayed with 50 µmol/L GA_3_ before *Agrobacterium* infiltration, a water spray was used as control. **a** Bright-field, **b** dark-field, **c** intensity of LUC bioluminescence quantified using Andor Solis image analysis software. Data are mean ± SEM of five biological replicates each with three technical repeats, 20 shoots were used in each technical repeat. Asterisks denote a significant *P* < 0.05

Encouraged by the high transient expression efficiency observed in our initial experiments, we next tested the applicability of our system in gene function/regulation studies in physiological contexts. As the floral repressor *RoKSN* is regulated by temperature and GA_3_ in roses [18], we chose to test whether these established responses could be detected and monitored by promoter activity in transiently transformed with the *pRoKSN:LUC* reporter construct consisting of the promoter of *RoKSN* cloned and fused to luciferase. As shown in Figs. 4 and 5, LUC activity was significantly suppressed by cold (4 °C) treatment and promoted by exogenous GA_3_ application, consistent with the literature [18]. This result demonstrates that our transient expression system is able to accurately detect transcriptional responses to different stimuli in rose shoots without detectable interference by *Agrobacterium* infection.

**Fig. 6:**
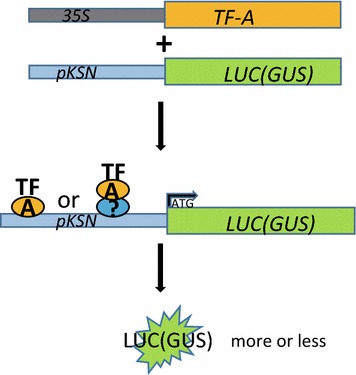
The schematic diagram of TF-promoter interaction screen. The specific TF coding gene under 35S promoter and *pKSN:LUC* constructs were co-infiltrated into rose shoots, the LUC expression will be activated or suppressed if interaction occurs between the TF and *pKSN*

**Fig. 7:**
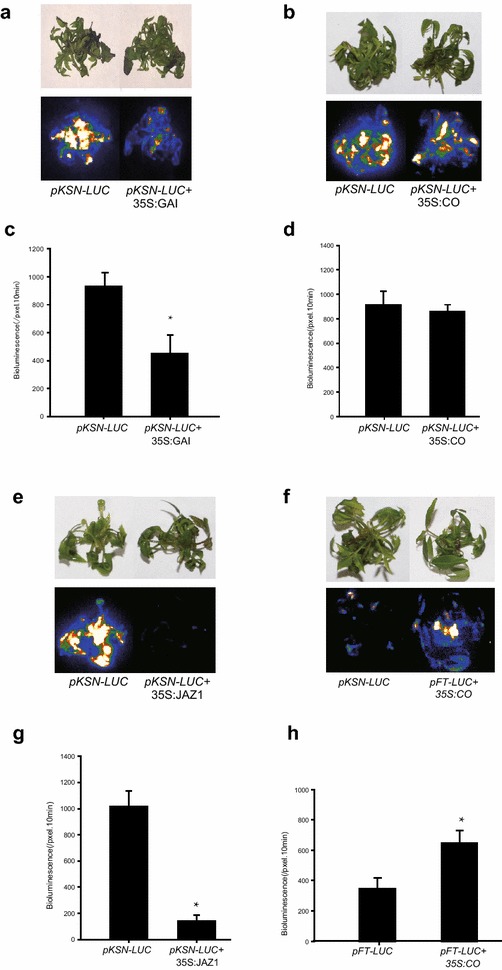
Interactions of TFs and *pRoKSN*. Representatives of transient expression assays in roses displayed by bright-field and dark-field (**a**, **b**, **e**) of leaves expressing *pKSN:LUC* alone, or together with different transcription factors. Intensity of LUC bioluminescence quantified using Andor Solis image analysis software (**c**, **d**, **g**). CO/*pFT:LUC* was used as a positive control (**e**, **h**). Data are mean ± SEM of five biological replicates each with three technical repeats, 20 shoots were used in each technical repeat. Asterisks denote a significant *P* < 0.05

Luciferase (LUC) is routinely used as report gene in the screening of interactions between TFs and promoters, with the LUC gene being driven by a promoter of interest and transiently co-expressed with a selected TF [19]. If the over-expressed TF can bind to the promoter of interest and drive the expression of LUC gene, the activities of LUC will be changed (Fig. 6). To further test the potential interaction of DELLA proteins and the promoter of *RoKSN* in roses, the abovementioned *pRoKSN:LUC* reporter construct was transiently co-expressed with the GA repressor *RoGAI* gene under the control of CaMV 35S promoter. As shown in Fig. 7, over-expression of *RoGAI* results in suppression of LUC activity, indicating direct or indirect regulation of the *RoKSN* promoter by *RoGAI*. This result also suggests a possible mechanism for the observed promotion of *pRoKSN:LUC* expression by GA; application of GA leads to DELLA (including GAI) protein degradation, thereby activating downstream gene expression. To eliminate the different levels of LUC activity was caused by discrepancy of plasmid amount, we analyzed the expression levels of inner standard Basta resistant gene carried by *pBGWL7* in both *pRoKSN:LUC* and *pRoKSN:LUC* + *RoGAI Agrobacterium*-infiltrated rose seedlings, the result clearly showed the consistency of Basta resistant gene expression in the two infiltrations (Additional file 3: Fig. S3), corresponding to the same amount of *pRoKSN:LUC* plasmid in both seedlings.

To further test the robustness of this method, three more combinations were selected for the binding analysis (Fig. 7c–h). Among them, the CO/FT model is very conserved in photoperiod induced flowering pathway within plant kingdom and acts as a well-established positive TF/promoter interaction control, although the basic level of *pRoFT:LUC* is relatively lower than *pRoKSN:LUC*. The RoJAZ1 significantly suppressed while RoCO has no affection on the activity of *pRoKSN:LUC*, reflecting the specificity of the present TF/promoter screening system.

### Protein to protein interaction analysis

**Fig. 8:**
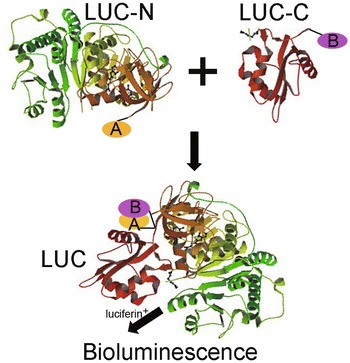
The principle of the split luciferase complementation assay for protein–protein interaction. Protein A and B were fused to N- or C-terminals of fly luciferase, LUC-N and LUC-C, respectively, and were co-infiltrated into rose shoots. The active luciferase enzyme will be reconstituted only when proteins interact, emitting light that can be visualized with a low-light imaging system

The split luciferase system has been widely used in protein–protein interaction studies. In this system, firefly luciferase is split into two fragments, NLuc and CLuc, which are then fused to proteins of interest. If the proteins of interest physically interact, the active luciferase enzyme is reconstituted and produces light that can be visualized with a low-light imaging system (the principle is illustrated in Fig. 8) [20, 21].

**Fig. 9:**
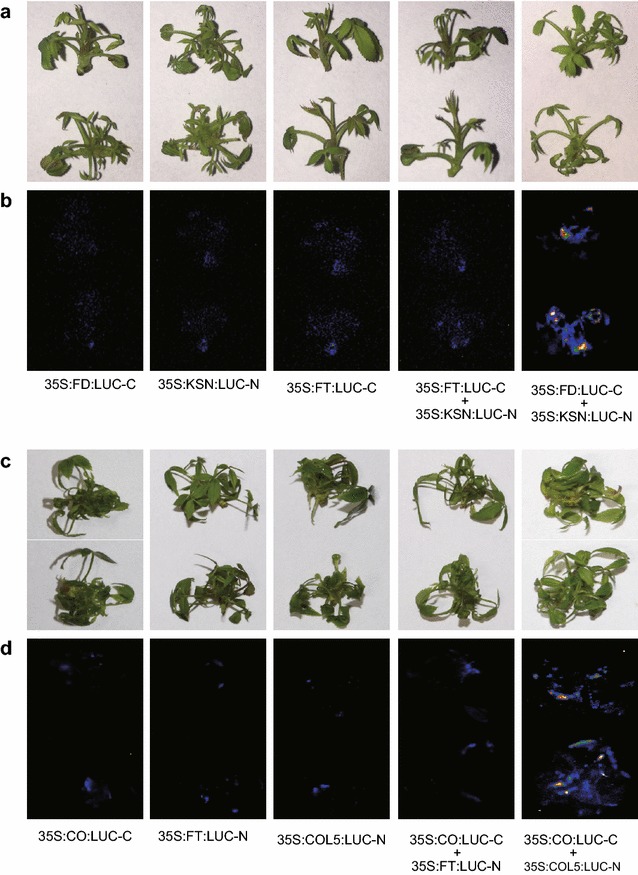
RoKSN and RoFD physically interact. Representatives of spilt luciferase complementation assays in rose shoots displayed by (**a**, **c**) bright-field, and (**b**, **d**) dark-field of leaves expressing *35S:RoKSN/35S:RoFT/35S:RoCOL5* fused to amino (N)-, and *35S:RoFD/35S:RoCO* fused to carboxy (C)-terminal fragment of luciferase. RoKSN and RoFD physically interact with each another (**b**), interactions of RoCO with RoFT and RoCOL5 were used as negative and positive control (**c**). This experiment was performed with five biological replicates each with three technical repeats, 20 shoots were used in each technical repeat

Because rose plants are less amenable for transient expression analysis, bimolecular fluorescence complementation (BiFC) studies for protein–protein interaction have often been conducted in *N. benthamian*a leaves via agroinfiltration. Here, we tested whether we could detect the previously established protein–protein interaction between RoKSN and RoFD by co-infecting rose shoots with *A. tumefaciens* strains carrying *35S:RoKSN:LUC*-*N* and *35S:RoFD:LUC*-*C*. The reconstituted LUC activity shown in Fig. 9 demonstrates the reliability of the rose transient expression system for BiFC studies. While the interactions of *35S:CO:LUC*-*C* with *35S:FT:LUC*-*N* and *35S:COL5:LUC*-*N* were used as controls. More interestingly, RoCO and RoCOL5 were firstly shown to interact with each another, implying COL5 is a potential flowering time regulator in rose plants via interfering CO binds to FT.

## Discussion

*Agrobacterium*-mediated transient transformation has been developed for a wide range of plants including Nicotiana, lettuce, tomato, and Arabidopsis [8–11]. In rose plants, petal infiltration using the VIGS system has been successfully achieved [12, 13]. Unlike leaf of Nicotiana, rose leaves are characterized by the stratum corneum and wax coat on the outermost layer of rose leaf and by the small leaf veins distributed parallel in roses. So far, these physiological features of mature rose leaves have made infiltration extremely difficult, even when a hole is made in the middle of leaf using a needle before infiltration. To overcome this barrier, we tested *Agrobacterium* infiltration in younger rose shoots of different sub-culture times finding that 3–4 weeks from sub-culturing is the best time for transformation (Fig. 1). Importantly, the optimized method conferred 100% infected seedlings with highly increased transient expression in shoots and also transient expression in roots of infected seedlings (Additional file 1: Fig. S1). Furthermore, in the present platform, no complicated inoculation media is required; even sterilized water free of any supplements can work very well. Previous studies have demonstrated the requirement for the vir regulon of the Ti plasmid for the transfer of oncogenes from *Agrobacterium tumefaciens* to plant cells, while the addition of AS to stimulate vir gene expression during the infection process is indispensable [15, 16], the present results didn’t confirm a positive effect of AS on LUC activities (Fig. 2), potentially due to the difference of *Agrobacterium* strain. The p19 protein of tomato bushy stunt virus (TBSV), generally used for PTGS prevention, is not necessary for the current system (Fig. 3), potentially due to species specificity of p19 function or a lack of PTGS in young rose seedlings after infiltration.

As a proof of concept, this transient transformation system was designed to analyze promoter activities, TF actions and protein–protein interactions in physiological contexts. Roses have two contrasting flowering types: once flowering (OF) and continuous flowering (CF). Continuous flowering of modern cultivated roses is controlled by a monogenic recessive locus called RB [22], is a homologue of the Arabidopsis *TERMINAL FLOWER 1* (*TFL1*) gene [14]. The rose *TFL1* gene was named *RoKSN*, which functions as a floral repressor. *RoKSN* was shown to interact with *RoFD* and protein–protein interaction experiments revealed that RoKSN and RoFT could compete with RoFD for repression and activation of blooming, respectively [14, 23]. Gibberellins (GA) regulate the floral transition of rose through promoting *RoKSN* transcription, likely through regulation of the *RoKSN* promoter, which contains GA-responsive cis-elements, whose deletion suppressed the response to GA in a heterologous system [18].

To further investigate the function of the gene in the above physiological processes, the promoter of *RoKSN* was selected and cloned into the pBGWL7 vector to make the *pKSN:LUC* cassette, which was then infiltrated into rose shoots with *Agrobacterium tumefaciens*. As shown in Fig. 5, the LUC activity was significantly enhanced by exogenous GA spraying, while suppressed when co-infiltrated with *Agrobacteria* containing a vector to constitutively express the GA repressor *GAI* gene (Fig. 7), suggesting the over-expressed RoGAI can potentially bind to the promoter of *RoKSN* and inhibit the expression of downstream LUC gene, consistent with the published results [18]. This rapid TF promoter interaction screening system based on luciferase activity provides a fast method to identify the exact TF(s) that drive the expression of a specific promoter, thus revealing promoter specificities for TFs even within the same family [19, 24].

The development of sensitive and versatile techniques to detect protein–protein interactions in vivo is important for understanding protein functions. To facilitate protein–protein interaction studies in plants, we adopted the luciferase complementation imaging assay. The LUC-N and LUC-C halves of the firefly luciferase reconstitute active luciferase enzyme only when fused to two interacting proteins, which can be visualized with a low-light imaging system [20, 21]. *RoKSN* and *RoFD* were cloned and fused to C-/N-terminal of firefly luciferase separately, then co-infiltrated into rose shoots, confirming a RoKSN-RoFD protein–protein interaction rather than a RoKSN-RoFT interaction (Fig. 9) [23]. Thus, the assay is simple and reliable in detection of protein–protein interactions in plants.

The abovementioned transiently transformed shoots can be transplanted to sub-culture medium containing selection antibiotics, following which, any the newly generated buds on the selection plates are potential stable transformation events, meaning that this transient expression system can be extended into a stable transformation option.

## Conclusion

Here we described a platform for *Agrobacterium*-mediated transient transformation in roses. With this system, no complicated inoculation media, supplements, or carefully tuned plant growth conditions are required. *Agrobacterium* culture suspended in MS medium, or even in sterilized water can be used. The most important factor impacting the transformation efficiency is seedling age, with 3–4 weeks from sub-culturing being the best time for transformation. This transient transformation system was tested to analyze promoter activities, TF actions and protein–protein interactions in physiological contexts, with the results clearly validating the robustness and efficiency of this system, thus providing a new option for gene function and signaling pathway investigation in roses.

## Methods

### Plant materials and growth conditions

In vitro propagated shoots of *R. chinensis* cv ‘Old Blush’ were used as starting material. They were repeatedly sub-cultured every 3–4 weeks on proliferation medium, which is MS + 1.5 mg/L 6-BA + 0.1 mg/L NAA + 30 g/L sucrose + 6.5 g/L agar, PH value is 5.75. Adventitious buds or shoot apexes were cut from maternal shoots and transferred into a 200 mL wide-mouth bottle with 30 mL propagation medium and kept growing. We find that at least 3 to 5-fold multiplication is easy obtained in 3–4 weeks (Additional file 4: Fig. S4) For root generation, half strength MS medium with 0.1 mg/L NAA, 30 g/L sucrose and 6.5 g/L agar was chosen. The incubation light intensity is 250 μmol/m^2^ S, photoperiod is 16 h light/8 h dark, and the room temperature is 25 °C. The young shoots with or without roots were used for *Agrobacterium* infiltration.

### *Agrobacterium* infection in rose shoots

A fresh single colony of *Agrobacterium* tumefaciens strain GV3101 carrying the gene of interest on a binary vector was selected to inoculate 3–5 mL of LB liquid medium containing appropriate antibiotics for shaking (220 rpm) overnight at 28 °C, then a large volume (50–200 mL) of medium inoculated with a 1:50 dilution of the overnight culture was incubated to logarithmic phase (OD_600_ = 0.5). *Agrobacterium* solutions were spun down at 5000×*g* for 10 min and the pellets were resuspended in desired infiltration buffer to OD_600_ = 0.2.

For vacuum infiltration, the standard medium (2 mM Na_3_PO_4_, 50 mM MES, 0.5% glucose, and 100 μM acetosyringone) for Nicotiana benthamiana infiltration, MS liquid medium with/without 100 μM acetosyringone, and sterilized water free of any supplements were used as infiltration buffer and compared. Sub-cultured rose shoots of different culture time were placed into the *Agrobacterium* suspension carrying gene of interest with/without p19 in a 200 mL wide-mouth bottle with about 50 mL *Agrobacterium* solution to ensure totally submerge the shoots, and infiltrated by vacuum at 0.5 MPa for 5 min in a sealed vacuum suction container (Additional file 5: Fig. S5). All of these procedures were performed in a laminar flow hood. After release of the vacuum, the shoots were washed by deionized water and kept on MS solid medium with 100 μM Timentin for 2–4 d before further LUC analysis. The p19 coding region from tomato bushy stunt virus was driven by 35S promoter in pBin19 binary vector [17] and was a gift from Professor Jiang Jiafu (Nanjing Agricultural University, PR, China).

### Plasmid construction for various analysis

For promoter and TF activities analysis, *pRoKSN* was amplified from the genomic DNA and the coding regions of the Della protein RoGAI was cloned from cDNA of *R. chinensis* cv ‘Old Blush’ using the listed primers (Additional file 6: Table S1) followed by cloning into pENTR-D-TOPO vector (Invitrogen). Subsequently, LR recombinations between the entry vectors and the binary vector pBGWL7, pFAST-R05 (http://www.psb.ugent.be/) were conducted to produce the *pRoKSN:LUC* and *35S:GAI:GFP* expression plasmids.

Protein–protein interaction analysis was performed as we described before [25], *p35S* was cloned from pEN-L4-p35S-R1, the coding regions of *RoKSN*, *RoFT*, *RoFD* were cloned from cDNA of *R. chinensis* cv ‘Old Blush’, and the firefly luciferase fragments amino acids 1–416 (LUC-N) and amino acids 398–550 (LUC-C) were cloned from the pBGWL7 vector using the primers listed in Additional file 6: Table S1. PCR primers were designed to include 22- and 25-bp attB and attBr sites followed by at least 18–25 bp of gene-specific sequences. The BP reactions were subsequently performed with PCR products and corresponding donor vector pDONR221 P1-P4, pDONR221 P4r-P3r, and pDONR221 P3-P2 to generate pENTR vectors L1-35S-L4, R4-RoKSN-R3, R4-RoFT-R3, L3-LUC-N-L2, and L3-LUC-C-L2. Multiple LR reactions were then executed to construct the expression plasmids *35S:RoKSN:LUC*-*N*, *35S:RoFT:LUC*-*C* and *35S:RoFD:LUC*-*C* by using pH7WG as destination vector (Outline of construction is shown in Additional file 7: Fig. S6).

### RNA extraction and gene expression anlysis

Total RNA of *Agrobacterium* infiltrated seedlings was extracted using the BioTeke Quick RNA isolation Kit (Cat. #: RP3301, BioTeke Corporation, Beijing, China) and 1 μg of high quality total RNA was reverse transcribed using the PrimeScriptTM RT reagent Kit (Cat. #: RR047A, TaKaRa, Dalian, China) according to the manufacturer’s instructions. Quantitative real-time PCR (qRT-PCR) was carried out to compare Basta gene expression levels in *pRoKSN:LUC* and *pRoKSN:LUC* plus *35S:GAI:GFP* infiltration rose seedlings, *RoTCPC* gene was used as references [26]. Three biological replicates with three technical replicates were performed for each experiment. The sequences of primers are available in Additional file 6: Table S1.

### Luciferase imaging

Luciferase imaging was performed as previously described using a CCD camera (Andor Technology) 96 h after infiltration [25, 27]. Images were acquired every 10 min for 60 min, and luciferase activity was quantified as mean counts per pixel per exposure time using Andor Solis image-analysis software (Andor Technology).

### Statistical analyses

To determine statistical significance, we employed Tukey’s honest significant difference (HSD) test. The difference was considered significant at *P* < 0.05.

## Additional files

**Additional file 1: Fig. S1.** The representatives 60 min time-course expression of *pKSN:LUC* in rose seedlings displayed by (A) dark-field and intensity of LUC bioluminescence (B) quantified using Andor Solis image analysis software. Data are mean ± SEM of five biological replicates each with three technical repeats, 20 shoots were used in each technical repeat.

**Additional file 2: Fig. S2.** The representatives of transient expression of *pKSN:LUC* in roots of rose seedlings displayed by (A) bright-field and (B) dark-field, the arrows indicated roots. This experiment was performed with five biological replicates each with three technical repeats, 20 shoots were used in each technical repeat (n = 20).

**Additional file 3: Fig. S3.** The expression levels of Basta resistant in rose seedlings infiltrated by Agrobacterium with *pKSN:LUC* and *pKSN:LUC* plus *35S:GAI*. The transcript levels were normalized to RoTCPC measured in the same samples. Data are mean fold differences ± SD of three biological replicates each with three technical repeats, Asterisks denote a significant *P* < 0.05.

**Additional file 4: Fig. S4.** The schematic diagram of shoot propagation. Adventitious buds or shoot apexes were cut in length of < 1 cm from maternal shoots and transferred into a 200 mL wide-mouth bottle with 30 mL propagation medium and kept growing for 3 to 4 weeks, then the shoots were collected and used for vacuum infiltration or sub-propagation materials.

**Additional file 5: Fig. S5.** The schematic illustration of vacuum infiltration. Rose shoots were placed on the bottom of a 200 mL wide-mouth bottle, and about 50 mL Agrobacterium suspension carrying genes of interest for infiltration were poured into the bottle to ensure totally submerge the shoots, then the wide-mouth bottle was moved in a vacuum suction container and the vacuum pump was started, the shoots were infiltrated by vacuum at 0.5 MPa for 3–5 min, all the procedures were performed in a laminar flow hood. After release of the vacuum, the shoots were washed by deionized water at least three times and kept on MS solid medium with 100 μM timentin for 2–4 d before further LUC analysis.

**Additional file 6: Table S1.** List of primers used.

**Additional file 7: Fig. S6.** The outline of multiple BP and LR reactions to generate expression vectors for protein-protein interaction assay. PCR primers were designed to include 22- and 25-bp attB and attBr sites followed by at least 18 to 25 bp of gene-specific sequences, then the BP reactions were performed with PCR products and corresponding donor vector pDONR221 P1-P4, pDONR221 P4r-P3r, and pDONR221 P3-P2 to generate pENTR vectors L1-35S-L4, R4-RoKSN-R3, R4-RoFT-R3, L3-LUC-N-L2, and L3-LUC-C-L2. Multiple LR reactions were subsequently executed to construct the expression plasmids 35S:RoKSN:LUC-N, 35S:RoFT:LUC-C and 35S:RoFD:LUC-C by using pB7WG as destination vector.
