# Supplementary figures and images for: An efficient transient expression system for gene function analysis in rose
Source: Plant Methods. 2017 Dec 22;13:116. doi: 10.1186/s13007-017-0268-1 (PMC5740963; doi:10.1186/s13007-017-0268-1)

A

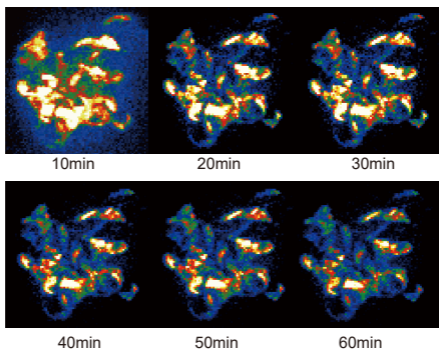

B

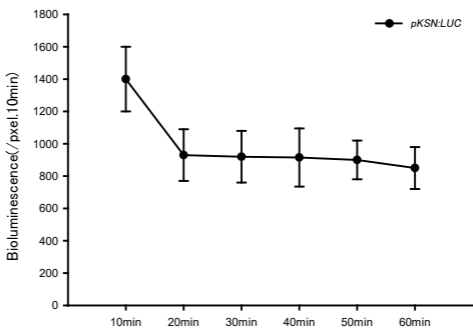

Supplement: Supplementary file 1 — Additional file 1: Fig. S1. The representatives 60 min time-course expression of pKSN:LUC in rose seedlings displayed by (A) dark-field and intensity of LUC bioluminescence (B) quantified using Andor Solis image analysis software. Data are mean ± SEM of five biological replicates each with three technical repeats, 20 shoots were used in each technical repeat. [file 13007_2017_268_MOESM1_ESM.pdf]

**A**

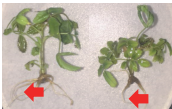

**B**

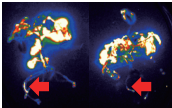

Supplement: Supplementary file 2 — Additional file 2: Fig. S2. The representatives of transient expression of pKSN:LUC in roots of rose seedlings displayed by (A) bright-field and (B) dark-field, the arrows indicated roots. This experiment was performed with five biological replicates each with three technical repeats, 20 shoots were used in each technical repeat (n = 20). [file 13007_2017_268_MOESM2_ESM.pdf]

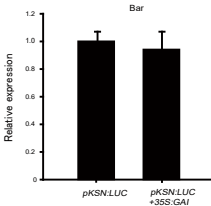

Supplement: Supplementary file 3 — Additional file 3: Fig. S3. The expression levels of Basta resistant in rose seedlings infiltrated by Agrobacterium with pKSN:LUC and pKSN:LUC plus 35S:GAI. The transcript levels were normalized to RoTCPC measured in the same samples. Data are mean fold differences ± SD of three biological replicates each with three technical repeats, Asterisks denote a significant P < 0.05. [file 13007_2017_268_MOESM3_ESM.pdf]

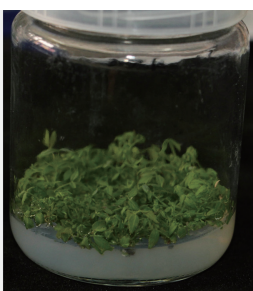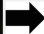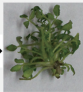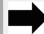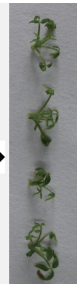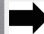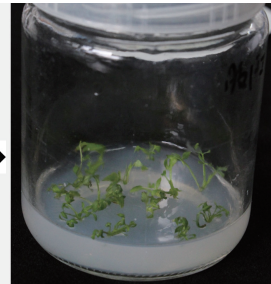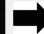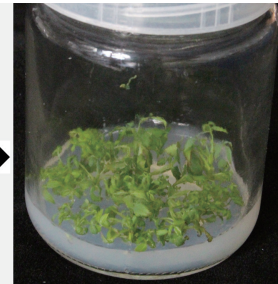

Supplement: Supplementary file 4 — Additional file 4: Fig. S4. The schematic diagram of shoot propagation. Adventitious buds or shoot apexes were cut in length of < 1 cm from maternal shoots and transferred into a 200 mL wide-mouth bottle with 30 mL propagation medium and kept growing for 3 to 4 weeks, then the shoots were collected and used for vacuum infiltration or sub-propagation materials. [file 13007_2017_268_MOESM4_ESM.pdf]

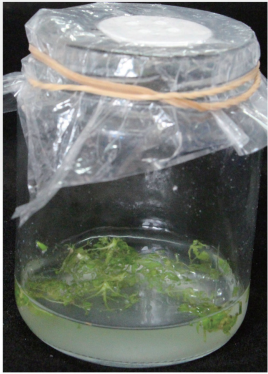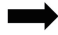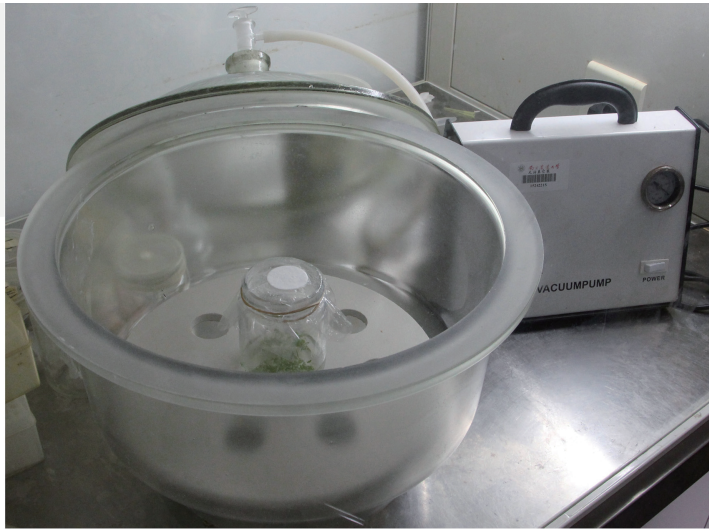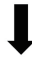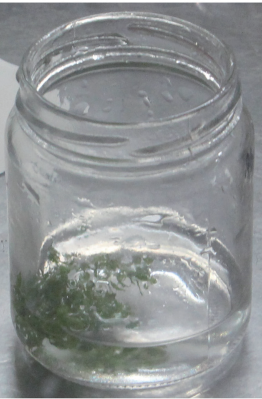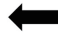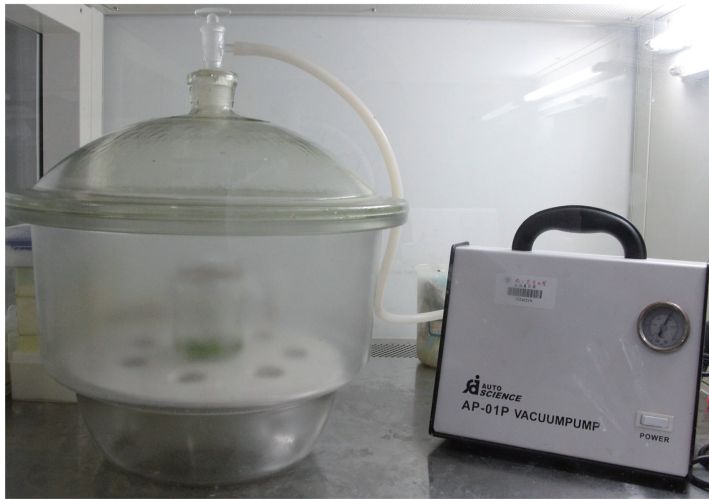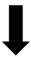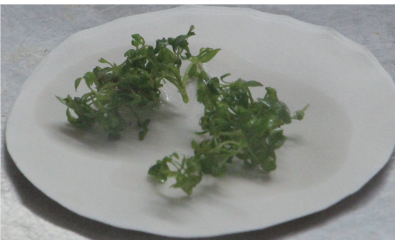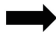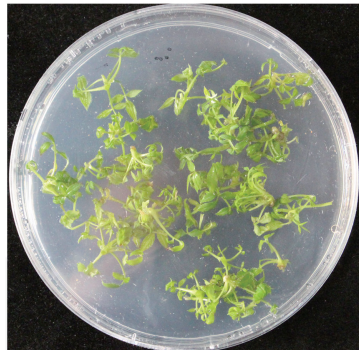

Supplement: Supplementary file 5 — Additional file 5: Fig. S5. The schematic illustration of vacuum infiltration. Rose shoots were placed on the bottom of a 200 mL wide-mouth bottle, and about 50 mL Agrobacterium suspension carrying genes of interest for infiltration were poured into the bottle to ensure totally submerge the shoots, then the wide-mouth bottle was moved in a vacuum suction container and the vacuum pump was started, the shoots were infiltrated by vacuum at 0.5 MPa for 3–5 min, all the procedures were performed in a laminar flow hood. After release of the vacuum, the shoots were washed by deionized water at least three times and kept on MS solid medium with 100 μM timentin for 2–4 d before further LUC analysis. [file 13007_2017_268_MOESM5_ESM.pdf]

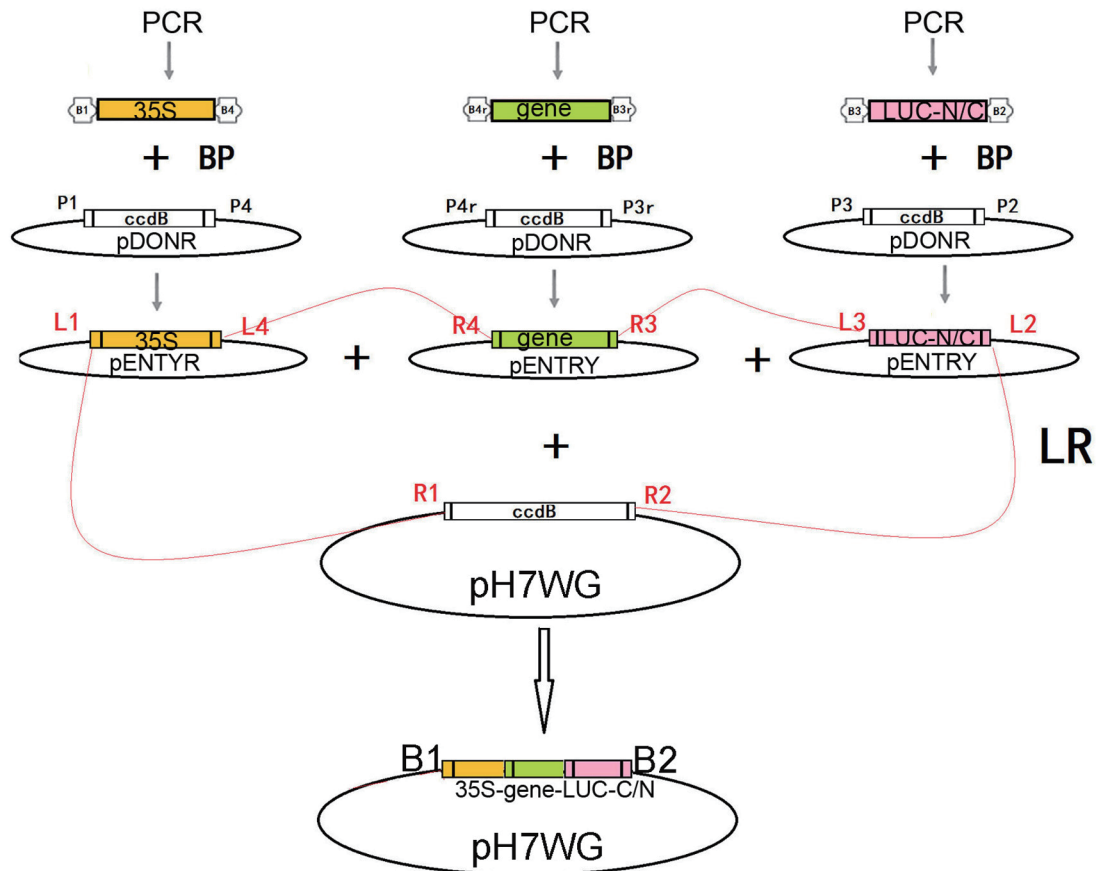

Supplement: Supplementary file 7 — Additional file 7: Fig. S6. The outline of multiple BP and LR reactions to generate expression vectors for protein-protein interaction assay. PCR primers were designed to include 22- and 25-bp attB and attBr sites followed by at least 18 to 25 bp of gene-specific sequences, then the BP reactions were performed with PCR products and corresponding donor vector pDONR221 P1-P4, pDONR221 P4r-P3r, and pDONR221 P3-P2 to generate pENTR vectors L1-35S-L4, R4-RoKSN-R3, R4-RoFT-R3, L3-LUC-N-L2, and L3-LUC-C-L2. Multiple LR reactions were subsequently executed to construct the expression plasmids 35S:RoKSN:LUC-N, 35S:RoFT:LUC-C and 35S:RoFD:LUC-C by using pB7WG as destination vector. [file 13007_2017_268_MOESM7_ESM.pdf]
