# Supplementary material for: An efficient transient expression system for gene function analysis in rose
Source: Plant Methods. 2017 Dec 22;13:116. doi: 10.1186/s13007-017-0268-1 (PMC5740963; doi:10.1186/s13007-017-0268-1)
Supplement: Supplementary file 6 — Additional file 6: Table S1. List of primers used. [file 13007_2017_268_MOESM6_ESM.docx]

**Additional file 6: Table S1. List of primers used**

| **Gene** | **Primers** | **Note** |
| --- | --- | --- |
| *pKSN* | F-CACCCATCCTATCAATCGTTA  R-GTACTCAAAAGCGAGAGAGGAGG | Cloning in entry vector pENTR-D- Topo |
| *RoGAI* | F-CACCATGGAGCCGTACGGCAATTCC  R- GACATGATTGGCACTATGAT | Cloning in entry vector pENTR-D- Topo |
| *RoKSN* | F-GGGGACAACTTTTCTATACAAAGTTGCCATGGCAAGAATTTCGGAACCTTTAG  R-GGGGACAACTTTATTATACAAAGTTGTGCGTCTTCTTGCTGCCGTTTCT | Amplify B4r-B3r fragment for BP reaction to generate R4-*RoKSN*-R3 |
| *RoFT* | F-GGGGACAACTTTTCTATACAAAGTTGCCATGCTTAAAACTATGCCTAGGGCT  R-GGGGACAACTTTATTATACAAAGTTGTTACTCTCCTTCCACCAGAGCCG | Amplify B4r-B3r fragment for BP reaction to generate R4-*RoFT*-R3 |
| *RoFD* | F-GGGGACAACTTTTCTATACAAAGTTGCCATGGAGGATGTGTGGAAAG  R-GGGGACAACTTTATTATACAAAGTTGTGCTACACAGAGTGTGCCCT | Amplify B4r-B3r fragment for BP reaction to generate R4-*RoFD*-R3 |
| Firefly luciferase-N | F-GGGGACAACTTTGTATAATAAAGTTGCCATGGAAGACGCCAAAAACATAAAGAAAG  R-GGGGACCACTTTGTACAAGAAAGCTGGGTATCAATCAAGGCGTTGGTCGCTTCC | Amplify B3-B2 fragment for BP reaction to generate L3-NLuc-L2 |
| Firefly luciferase-C | F-GGGGACAACTTTGTATAATAAAGTTGCCATGTCCGGTTATGTAAACAATCCG  R-GGGGACCACTTTGTACAAGAAAGCTGGGTATTACACGGCGATCTTTCCGCCC | Amplify B3-B2 fragment for BP reaction to generate L3-CLuc-L2 |
| p35S | F-GGGGACAAGTTTGTACAAAAAAGCAGGCTCCATTTAGGTGACACTATAGAATACTC  R-GGGGACAACTTTGTATAGAAAAGTTGGGTGTGATATCACTAGTGCGGCCGCT | Amplify B1-B4 fragment for BP reaction to generate L1-p35S-L4 |
| Bar | F-ATGACAGCGACCACGCTCTT, R-AAGGCACGCAACGCCTACGACT | q-RT |
| RcTCTP | F-TTGGTCTTTGCCTACTACAAAGAGG, R-AAGCCAGTTGCTACTTCTTAGCACT | Reference for q-RT |
